# Supplementary material for: An open-source framework for large-scale, flexible evaluation of biomedical text mining systems
Source: J Biomed Discov Collab. 2008 Jan 29;3:1. doi: 10.1186/1747-5333-3-1 (PMC2276192; doi:10.1186/1747-5333-3-1)
Supplement: Additional file 1 — Evaluation platform source code. The additional file contains the software evaluation framework discussed in this paper. The most current release can be downloaded from SourceForge.net [1]. See the accompanying README file for instructions on installation and use. Also included in the distribution is a collection of UIMA wrappers for some commonly used BioNLP tools and annotated corpora. [file 1747-5333-3-1-S1.zip › bionlp_uima_repository-1.0.1/docs/api/src-html/edu/uchsc/ccp/uima/annotation/CCPDocumentSection_Type.html]

```
001    
002    /* First created by JCasGen Tue Aug 28 16:44:10 MDT 2007 */
003    package edu.uchsc.ccp.uima.annotation;
004    
005    import org.apache.uima.cas.Feature;
006    import org.apache.uima.cas.FeatureStructure;
007    import org.apache.uima.cas.Type;
008    import org.apache.uima.cas.impl.CASImpl;
009    import org.apache.uima.cas.impl.FSGenerator;
010    import org.apache.uima.cas.impl.FeatureImpl;
011    import org.apache.uima.cas.impl.TypeImpl;
012    import org.apache.uima.jcas.JCas;
013    import org.apache.uima.jcas.JCasRegistry;
014    
015    /** The Document Section annotation allows for an entire document section to be annotated as such, and stores section-specific information such as the section identifier.
016     * Updated by JCasGen Tue Aug 28 16:44:10 MDT 2007
017     * @generated */
018    public class CCPDocumentSection_Type extends CCPDocumentAnnotation_Type {
019      /** @generated */
020      protected FSGenerator getFSGenerator() {return fsGenerator;}
021      /** @generated */
022      private final FSGenerator fsGenerator = 
023        new FSGenerator() {
024          public FeatureStructure createFS(int addr, CASImpl cas) {
025                             if (CCPDocumentSection_Type.this.useExistingInstance) {
026                               // Return eq fs instance if already created
027                         FeatureStructure fs = CCPDocumentSection_Type.this.jcas.getJfsFromCaddr(addr);
028                         if (null == fs) {
029                           fs = new CCPDocumentSection(addr, CCPDocumentSection_Type.this);
030                               CCPDocumentSection_Type.this.jcas.putJfsFromCaddr(addr, fs);
031                               return fs;
032                         }
033                         return fs;
034            } else return new CCPDocumentSection(addr, CCPDocumentSection_Type.this);
035              }
036        };
037      /** @generated */
038      public final static int typeIndexID = CCPDocumentSection.typeIndexID;
039      /** @generated 
040         @modifiable */
041      public final static boolean featOkTst = JCasRegistry.getFeatOkTst("edu.uchsc.ccp.uima.annotation.CCPDocumentSection");
042     
043      /** @generated */
044      final Feature casFeat_sectionID;
045      /** @generated */
046      final int     casFeatCode_sectionID;
047      /** @generated */ 
048      public int getSectionID(int addr) {
049            if (featOkTst && casFeat_sectionID == null)
050          jcas.throwFeatMissing("sectionID", "edu.uchsc.ccp.uima.annotation.CCPDocumentSection");
051        return ll_cas.ll_getIntValue(addr, casFeatCode_sectionID);
052      }
053      /** @generated */    
054      public void setSectionID(int addr, int v) {
055            if (featOkTst && casFeat_sectionID == null)
056          jcas.throwFeatMissing("sectionID", "edu.uchsc.ccp.uima.annotation.CCPDocumentSection");
057        ll_cas.ll_setIntValue(addr, casFeatCode_sectionID, v);}
058        
059      
060     
061      /** @generated */
062      final Feature casFeat_sectionLength;
063      /** @generated */
064      final int     casFeatCode_sectionLength;
065      /** @generated */ 
066      public int getSectionLength(int addr) {
067            if (featOkTst && casFeat_sectionLength == null)
068          jcas.throwFeatMissing("sectionLength", "edu.uchsc.ccp.uima.annotation.CCPDocumentSection");
069        return ll_cas.ll_getIntValue(addr, casFeatCode_sectionLength);
070      }
071      /** @generated */    
072      public void setSectionLength(int addr, int v) {
073            if (featOkTst && casFeat_sectionLength == null)
074          jcas.throwFeatMissing("sectionLength", "edu.uchsc.ccp.uima.annotation.CCPDocumentSection");
075        ll_cas.ll_setIntValue(addr, casFeatCode_sectionLength, v);}
076        
077      
078     
079      /** @generated */
080      final Feature casFeat_sectionName;
081      /** @generated */
082      final int     casFeatCode_sectionName;
083      /** @generated */ 
084      public String getSectionName(int addr) {
085            if (featOkTst && casFeat_sectionName == null)
086          jcas.throwFeatMissing("sectionName", "edu.uchsc.ccp.uima.annotation.CCPDocumentSection");
087        return ll_cas.ll_getStringValue(addr, casFeatCode_sectionName);
088      }
089      /** @generated */    
090      public void setSectionName(int addr, String v) {
091            if (featOkTst && casFeat_sectionName == null)
092          jcas.throwFeatMissing("sectionName", "edu.uchsc.ccp.uima.annotation.CCPDocumentSection");
093        ll_cas.ll_setStringValue(addr, casFeatCode_sectionName, v);}
094        
095      
096     
097      /** @generated */
098      final Feature casFeat_subSections;
099      /** @generated */
100      final int     casFeatCode_subSections;
101      /** @generated */ 
102      public int getSubSections(int addr) {
103            if (featOkTst && casFeat_subSections == null)
104          jcas.throwFeatMissing("subSections", "edu.uchsc.ccp.uima.annotation.CCPDocumentSection");
105        return ll_cas.ll_getRefValue(addr, casFeatCode_subSections);
106      }
107      /** @generated */    
108      public void setSubSections(int addr, int v) {
109            if (featOkTst && casFeat_subSections == null)
110          jcas.throwFeatMissing("subSections", "edu.uchsc.ccp.uima.annotation.CCPDocumentSection");
111        ll_cas.ll_setRefValue(addr, casFeatCode_subSections, v);}
112        
113       /** @generated */
114      public int getSubSections(int addr, int i) {
115            if (featOkTst && casFeat_subSections == null)
116          jcas.throwFeatMissing("subSections", "edu.uchsc.ccp.uima.annotation.CCPDocumentSection");
117        if (lowLevelTypeChecks)
118          return ll_cas.ll_getRefArrayValue(ll_cas.ll_getRefValue(addr, casFeatCode_subSections), i, true);
119        jcas.checkArrayBounds(ll_cas.ll_getRefValue(addr, casFeatCode_subSections), i);
120            return ll_cas.ll_getRefArrayValue(ll_cas.ll_getRefValue(addr, casFeatCode_subSections), i);
121      }
122       
123      /** @generated */ 
124      public void setSubSections(int addr, int i, int v) {
125            if (featOkTst && casFeat_subSections == null)
126          jcas.throwFeatMissing("subSections", "edu.uchsc.ccp.uima.annotation.CCPDocumentSection");
127        if (lowLevelTypeChecks)
128          ll_cas.ll_setRefArrayValue(ll_cas.ll_getRefValue(addr, casFeatCode_subSections), i, v, true);
129        jcas.checkArrayBounds(ll_cas.ll_getRefValue(addr, casFeatCode_subSections), i);
130        ll_cas.ll_setRefArrayValue(ll_cas.ll_getRefValue(addr, casFeatCode_subSections), i, v);
131      }
132     
133    
134    
135    
136      /** initialize variables to correspond with Cas Type and Features
137            * @generated */
138      public CCPDocumentSection_Type(JCas jcas, Type casType) {
139        super(jcas, casType);
140        casImpl.getFSClassRegistry().addGeneratorForType((TypeImpl)this.casType, getFSGenerator());
141    
142     
143        casFeat_sectionID = jcas.getRequiredFeatureDE(casType, "sectionID", "uima.cas.Integer", featOkTst);
144        casFeatCode_sectionID  = (null == casFeat_sectionID) ? JCas.INVALID_FEATURE_CODE : ((FeatureImpl)casFeat_sectionID).getCode();
145    
146     
147        casFeat_sectionLength = jcas.getRequiredFeatureDE(casType, "sectionLength", "uima.cas.Integer", featOkTst);
148        casFeatCode_sectionLength  = (null == casFeat_sectionLength) ? JCas.INVALID_FEATURE_CODE : ((FeatureImpl)casFeat_sectionLength).getCode();
149    
150     
151        casFeat_sectionName = jcas.getRequiredFeatureDE(casType, "sectionName", "uima.cas.String", featOkTst);
152        casFeatCode_sectionName  = (null == casFeat_sectionName) ? JCas.INVALID_FEATURE_CODE : ((FeatureImpl)casFeat_sectionName).getCode();
153    
154     
155        casFeat_subSections = jcas.getRequiredFeatureDE(casType, "subSections", "uima.cas.FSArray", featOkTst);
156        casFeatCode_subSections  = (null == casFeat_subSections) ? JCas.INVALID_FEATURE_CODE : ((FeatureImpl)casFeat_subSections).getCode();
157    
158      }
159    }
160    
161    
162    
163
```
